# Photoluminescence and Band Alignment of Strained GaAsSb/GaAs QW Structures Grown by MBE on GaAs

**DOI:** 10.3390/ma3031497

**Published:** 2010-02-26

**Authors:** Yuri G. Sadofyev, Nigamananda Samal

**Affiliations:** Trion Technology, Tempe, Arizona 85281, USA; E-Mail: yuri@triontech.com (Y.G.S.)

**Keywords:** molecular beam epitaxy, quantum wells, antimonide, laser diodes

## Abstract

An in-depth optimization of growth conditions and investigation of optical properties including discussions on band alignment of GaAsSb/GaAs quantum well (QW) on GaAs by molecular beam epitaxy (MBE) are reported. Optimal MBE growth temperature of GaAsSb QW is found to be 470 ± 10 °C. GaAsSb/GaAs QW with Sb content ~0.36 has a weak type-II band alignment with valence band offset ratio Q_V_ ~1.06. A full width at half maximum (FWHM) of ~60 meV in room temperature (RT) photoluminescence (PL) indicates fluctuation in electrostatic potential to be less than 20 meV. Samples grown under optimal conditions do not exhibit any blue shift of peak in RT PL spectra under varying excitation.

## 1. Introduction

During last decade, GaAs_1-x_Sb_x_/GaAs material system—preferably near 0.36 mole fraction—has been keenly investigated due to its potential for commercial manufacturing of monolithic vertical cavity surface emitting lasers (VCSELs), which operate near 1.3 μm fiber-optic telecommunication spectrum. Typically, the devices are based on combination of GaAsSb/GaAs QW and AlGaAs/GaAs distributed Bragg reflectors (DBR). In spite of reported intense room temperature (RT) photoluminescence (PL), GaAsSb material system has not yet demonstrated enough output power in 1.3 μm VCSELs to make them commercially viable [1,2]. Reasons are many. Difficulty in growing high quality QW active material, due to lattice mismatch of 7.8% between GaSb and GaAs [3], is believed to be the prime reason. Moreover, a miscibility gap [4] in forming GaAsSb alloy, *i.e.*, additional complications in growing GaAsSb with Sb mole fraction between 0.25 and 0.7, adds to the problem.

Chang *et al.* have demonstrated growth of GaAsSb over the entire composition of Sb [5]. The composition was controlled by the growth temperature, chemical species, flux ratios, and surface diffusion at the vacuum/solid interface. However, lack of detailed information on crystalline structure of GaAsSb epilayers makes it difficult to clearly understand non-uniform spatial composition of Sb as a result of miscibility gap. Evidence of partial spinodal decomposition has been clearly observed by transmission electron microscopy [6]. The growth within the miscibility gap is attributed to the 'latching effect' of substrate-induced stabilization [7], in which the strain energy is sufficient to prevent spinodal decomposition of the metastable phase.

There is no clear consensus yet on the type of band alignment in GaAsSb/GaAs system. Some researchers insist on type-II band alignment with valence band offset ratio, Q_V_ = ΔE_V_/ΔE_g_, to lie somewhere in between 1.05 and 2.1 [8,9,10,11]. Some insist a flat conduction band [12,13], for which Q_V_ = 1, while others [14] insist a type-I band alignment, for which Q_V_ < 1. Johnson *et al.* [15] have shown that the type of band lineup for strained GaAs_1-x_Sb_x_/GaAs QW is a function of Sb mole fraction and it changes from type-I for x < 0.34 to type-II for x > 0.34. Despite lack of clear consensus, one can safely suppose that band lineup for GaAsSb/GaAs QW would be somewhere between weak type-II and weak type-I for Sb molar fraction of ~0.36. In other words, GaAsSb/GaAs QW can be considered as a system with strongly localized holes and weakly localized electrons, especially at relatively higher temperature, *i.e.*, 300 K or more.

The PL under varying temperatures and excitation intensities usually provides evidence to establish theoretical models for characterizing QW materials. RT PL spectra of GaAsSb/GaAs QW, usually, are fairly wide, *i.e.*, from 60 to 140 meV [9,15,16] and PL peak, usually, demonstrates blueshift with increase in the excitation intensity. The widening of PL is typically attributed to type-II band lineup [8,17] and fluctuations in electrostatic potential inside QW, later as a result of material disorder. Blueshift of PL peak is typically attributed to electrostatic band-bending and band-filling of localized states, caused by fluctuations of electrostatic potential in QW, as a consequence of interface roughness or spatial non-uniformity in Sb alloy. There are several reasons for spatial non-uniformity in Sb alloy in GaAsSb layer grown on GaAs. A miscibility gap and Sb segregation caused by built-in mechanical strain are most important among them. Transmission electron microscopy confirms non-uniform distribution of Sb in QW [18], at least in determinate conditions of epitaxial growth. One must, however, pay close attention to the role of technological limitations—which researchers often tend to overlook—in epitaxial growth, *i.e.*, MBE, which might contribute to observed physical effects before hypothesizing models for intrinsic optical properties of the QW. Unfortunately, limited work has been published to date in terms of optimization of growth conditions of GaAsSb on GaAs [15].

In this work, an in-depth optimization of growth conditions of GaAsSb on GaAs (1 0 0) by MBE and investigation of optical properties of GaAsSb/GaAs QW, including discussions on possibility of different types of band alignment—through various designs of barrier and cladding layers—are discussed.

## 2. Experimental Details

The QW structures are grown by MBE on EPINEAT (RIBER) chamber, equipped with solid-source cells. The Ga and Al cells—manufactured by E-SCIENCE—are common effusion cells with carbon and PBN crucibles respectively. The valved cracker cells—manufactured by APPLIED EPI—are used for As and Sb evaporation. Cracking zone of the arsenic cell, operating at 990 °C, provides molecular flux of As dimers. A mix of antimony monomers and dimers is generated by corrosive series valved cracker cell at cracking zone temperature of 1060 °C. Standard calibration procedure of equivalent beam pressure (EBP) measurement for group III and group V elements are usually done by means of beam flux monitor (BFM) at the beginning of each growth campaign. Curves for EBP versus cell temperatures of gallium and aluminum are converted to curves for growth rates of GaAs and AlAs versus cell temperature. The growth rates are typically obtained from spectra of optical reflectivity on grown GaAs/AlAs double Bragg reflector structures with 1λ cavity. By fitting experimental spectra to theoretically simulated spectra, real growth rates for GaAs and AlAs are determined with an error within a few tenth of a percent.

Observing transition from group-V rich to group-III rich surface through RHEED gives an accurate idea on absolute V/III flux ratio (F_V/III_). The calibration is based on RHEED patterns on GaAs (1 0 0) epitaxial layer surface. Sharp transition from As-rich intermediate (5 × 1) to Ga-rich (4 × 2) surface reconstruction at 580 °C serves as a bench mark for V/III = 1:1 ratio at determinate Ga(Al)As growth rate. Degradation of (3 × 1) GaSb (1 0 0) surface reconstruction at substrate temperature 510 °C has been chosen as benchmark for Sb/Ga ratio equal to 1:1.

W-Re (Type C) thermocouple (TC) based automatic control is used for monitoring and controlling substrate temperature. Parallel control of substrate temperature through heated view port is provided by infrared (IR) pyrometer in manual mode. All temperatures noted below are measured by IR pyrometer. TC and GaAs wafer have different thermal inertia. Thus, profile of growth temperature is usually adjusted to obtain unchanged pyrometer readings during QW growth.

All samples are grown on 3"n^+^ -GaAs (1 0 0) ± 0.1° wafers at GaAs growth rate of 15 nm/min. First set of single QW GaAsSb samples with thick GaAs barrier layers was intended to optimize growth conditions and characterize intrinsic properties of the QW. The QW structure is as follows: A 300 nm GaAs buffer was followed by a sequence of 50 nm Al_0.3_Ga_0.7_As cladding, 50 nm GaAs barrier, 7 nm GaAsSb QW, 50 nm GaAs barrier and 50 nm Al_0.3_Ga_0.7_As cladding and terminated by a 30 nm GaAs cap layer. GaAs buffer and AlGaAs layers were grown at substrate temperature of 580 °C with As/III ratio of 1.3. Growth temperature of QW was varied from 460 to 510 °C. Change of temperature was done either during growth of GaAs barrier or AlGaAs cladding layers depending on the difference of temperature to be realized at the quantum well.

Sticking factors of group V species are seen to be less than unity in MBE. Preferential order of incorporation, from high to low, among determinate species is III-V_A_-V_B_ alloys, *i.e.*, group-III elements get the highest preference of incorporation. The degree of preference, however, is affected by both substrate temperature and V/III ratio. For instance, As_2_ is preferentially incorporated relative to Sb at substrate temperature 500 °C, while at 450 °C the preference is just opposite. In our experiment, Sb/III ratio of 0.42–0.45 was used to obtain RT PL peak at ~(1280–1300) nm. As/Ga ratio for GaAsSb growth was kept equal to unity for all samples. Growth rate of 15 nm/min for GaAs was typical for most of the samples except a few, which were grown at relatively lower rate, *i.e.*, 5 nm/min.

High power laser diode, emitting 250 mW power at λ = 671 nm, and monochromator (Applied Optronics Corp.) with InGaAs photodetector were used for PL measurements. Typical spot size of laser beam in PL measurement was ~1 mm in diameter.

## 3. Results and Discussion

**Figure 1:**
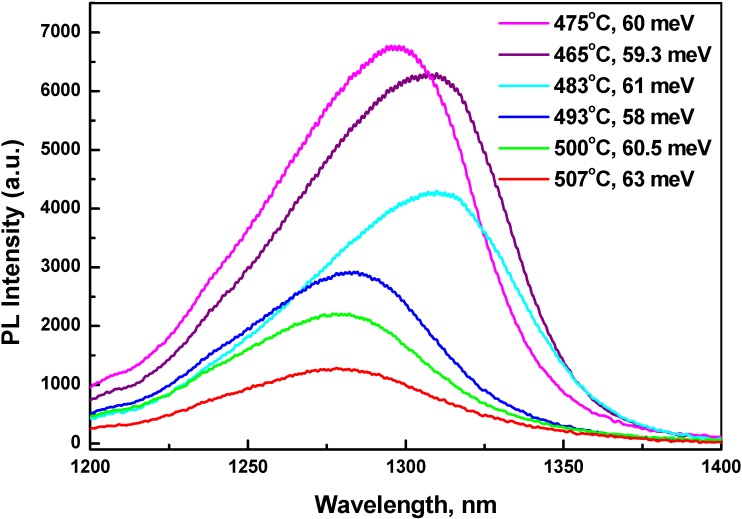
RT PL spectra from six single QW structures to optimize growth conditions. High power diode laser (Applied Optronics) emitting 250 mW at 671 nm was used for PL excitation. Growth temperatures and FWHM of PL lines are indicated against each curve.

RT PL spectra, growth temperatures (°C) and FWHM (meV) of the first set of QW structures are shown in Figure 1. PL peaks are observed between 1275 and 1310 nm with their tendency to redshift with decrease in substrate temperature. The redshift of PL is attributed to higher sticking coefficient of Sb at lower temperature. The PL intensity increases and FWHM decreases with decrease in substrate temperature from 507 to 465 °C. PL FWHM are within 58–63 meV for all samples. Usually, PL intensity drops for growth at lower temperature [15] as a result of unintentional doping and increase in point defects. Based on observed PL intensity and FWHM, optimal growth temperature of GaAsSb/GaAs QW is determined to be 470 ± 10°C.

**Table 1:** Fluctuations in electrostatic potential distribution *(δE)* of GaAsSb/GaAs QW versus growth temperature T_Sub_ of the QW.

T_Sub_, °C	465	475	483	493	500	507
*δE*, *m*eV	16.2	16.4	17.3	23.4	28.5	29.8

Dinu *et al.* [9] have shown that low-energy slope of PL spectrum is independent of temperature, and thus attributed to an exponential tail of localized states, due possibly to fluctuations in electrostatic potential in QW. The high energy slope of the PL spectrum corresponds to a decay, exp( *-E/kT*_0_), with an equivalent temperature (*T*_0_) [8]. The potential fluctuations are estimated by fitting the low-energy tail with a density of states of the form *g*(E) *∝* 1+tanh[(*E* *− E*_0_*)/δE*], where *E*_0_ is the center and *δE* is the width of the distribution and denote magnitude of potential fluctuations. In their approximation, *δE* is ~1.54 times the low-energy slope. We have calculated potential fluctuations (*δE*, meV) in similar way for all the samples shown in Figure 1 and are noted in Table 1.

**Figure 2:**
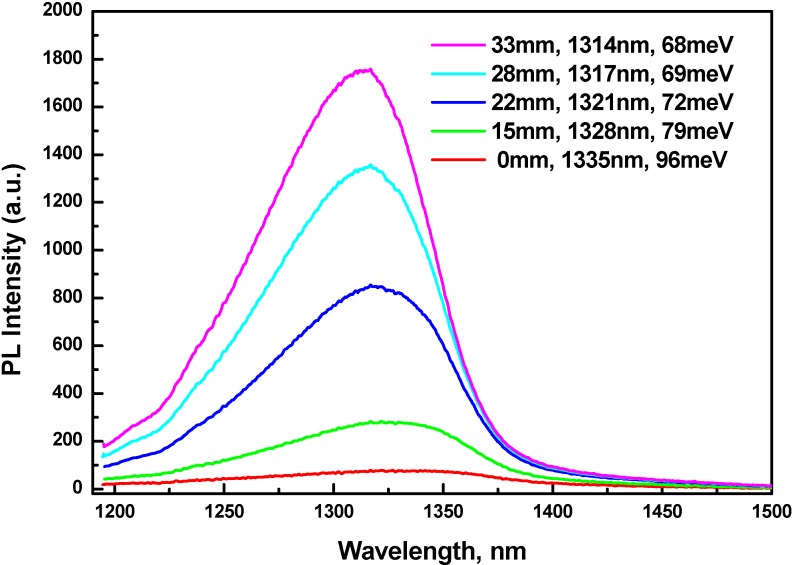
RT PL spectra of a GaAsSb/GaAs single QW structure at different distance from center of wafer.

Flux distribution of As and Sb is not uniform in space inside the MBE chamber. The wafer rotates during the MBE growth. As a result, a QW PL line position varies from center (maximal wavelength) to edge of the wafer (minimal wavelength). Variation in PL line position was ~20 nm for all our structures. It allows us to observe degradation of the PL line at high (> 0.36) molar fraction of Sb in QW. A family of PL lines—measured at different distance from center of the wafer marked in millimeters—from one GaAsSb/GaAs QW structure grown at 464 °C, is shown in Figure 2. PL peaks (in nanometers) and FWHM (in meV) are shown against the spectra as well. PL intensity decreases by one order of magnitude with peak position changing from 1315 to 1335 nm. This substantial reduction of PL intensity is attributed to misfit dislocations generated as a result of partial biaxial strain relaxation. Besides, FWHM increases with Sb content. The potential fluctuations, (*δE,* meV), for curves shown in Figure 2 range from 18.8 to 34.8 meV. Widening of FWHM is attributed to fluctuations in electrostatic potential as a result of miscibility gap. We can conclude that maximum wavelength one could reach, with fairly intensive PL, for a 7 nm thick GaAsSb/GaAs QW structure without strain compensation, is approximately 1330 nm.

**Figure 3:**
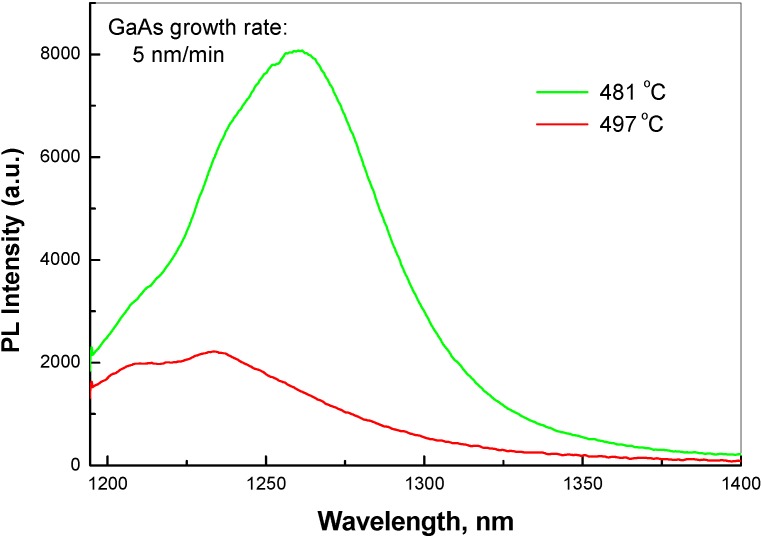
RT PL spectra from two single QW structures grown at low (5 nm/min) growth rate and at different temperatures.

RT PL spectra of two QW structures, grown at low growth rate (5 nm/min) and at different temperature, are shown in Figure 3. For these samples, Sb/Ga ratio was not adjusted to position the PL line around 1280–1300 nm. At higher growth temperature, a tendency of splitting the PL line in to two peaks is clearly observed. It could represent transition from a kinetics limited growth to a growth, substantially contributed by thermodynamics. The thermodynamics component might be responsible for alloy fluctuations in GaAsSb caused by alloy decomposition within the miscibility gap.

Table 1, Figure 1 and Figure 3 show that the disorder in QW material increases with increase in growth temperature. In optimum conditions of MBE growth, PL still remains wide, *i.e.*, FWHM of ~60 meV for peaks centered between 1275 and 1310 nm.

**Figure 4:**
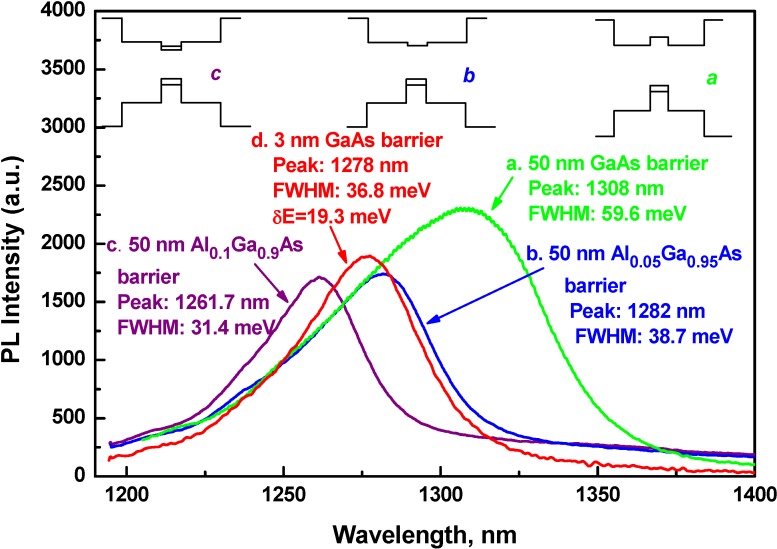
RT PL spectra from three single QW structures with 50 nm barrier layers consisting of GaAs (curve a), Al_0.05_Ga_0.95_As (curve b), and Al_0.1_Ga_0.9_As (curve c). RT PL of one with thin GaAs (3nm) barrier and single QW is shown in curve 'd' for comparison with those with thicker barriers, showing blue shift of its peak and narrower linewidth with respect to samples of either curve 'a' or curve 'b'. Insets: schematics of QW band alignment for the curves 'a', 'b' and 'c'; Inset *a* corresponds to QW with 50 nm GaAs barrier showing a type-II band lineup; Inset *b* corresponds to QW with 50 nm Al_0.05_Ga_0.95_As barrier showing a weak type-I band lineup with almost flat conduction band; Inset *c* corresponds to 50 nm Al_0.1_Ga_0.9_As barrier showing a stronger type-I band lineup.

However, it must be emphasized here that broadening of PL line should not be attributed to potential fluctuations only. Rather, the band lineup plays a bigger role. To prove this, a second set of four samples was grown with 50 nm GaAs barriers replaced by 50 nm Al_y_Ga_1-y_As barriers with varying aluminum content *y* = 0.05, 0.10, 0.15 and 0.20. Other growth conditions and rest of structural cross-section were kept unchanged. Strong change in RT PL was observed up to aluminum content y = 0.1. Increase in aluminum content above y = 0.1 does not change the shape and position of PL line. Figure 4 shows PL spectra from three single QW structures with barrier layers consisting of GaAs (curve a), Al_0.05_Ga_0.95_As (curve b) and Al_0.1_Ga_0.9_As (curve c). The barrier layer Al content, position of PL peak and FWHM are noted in the figures as well. Increase in Al content causes blueshift and narrowing of PL lines. Blueshift of 'b' and 'c' relative to 'a' are 19.2 and 34.8 meV respectively. FWHM values—59.6, 38.7 and 31.4 meV for 'a', 'b', and 'c' respectively—decrease with increase in Al content. All three GaAsSb QW samples were grown at 464 °C. Thus, reduction of linewidth, as seen in Figure 4, can not solely be attributed to better uniformity in QW material, allegedly caused by lesser spatial potential fluctuation and spinodal GaAsSb decomposition.

The above observations can be reasonably explained if we suppose that a transition from type-II to type-I band alignment takes place as a result of increase in Al content in barrier layers of the QW. In the insets of Figure 4, schematics of the energy band alignment for the curves 'a', 'b' and 'c' are shown. Inset *a* corresponds to QW with GaAs barriers having a type-II band lineup. Direct optical transitions in GaAsSb and indirect transitions between GaAs and GaAsSb layers are possible in this case. Combination of transitions is expected to result in distortion and broadening of PL. Replacement of GaAs barrier layers by Al_y_Ga_1-y_As would tend to form type-I band lineup with increasing aluminum content. This is illustrated in inset *b* and inset *c*. In these circumstances, PL FWHM of 31.4 meV (curve 'c' in Figure 4) can be accepted as a characteristic parameter for quality of QW material because RT PL is relatively narrow compared to others. In this situation, conduction band offset of GaAs/GaAsSb QW, *i.e.*, QW of curve 'a', can be estimated as a difference in FWHMs between curves 'a' (59.6 meV) and 'c' (31.4 meV), *i.e.*, between PL lines formed by superposition of both direct and indirect channels of optical transition (curve 'a') and the one with only direct optical transition (curve 'c'). The difference, *i.e.*, the conduction band offset, is 28.2 meV. PL peak of curve 'a' is at 1308 nm. Strained GaAsSb/GaAs [12] QW with Sb content ~0.37 radiates at wavelength 1308 nm at RT. An equation for variation in band gap difference ΔE_g_(*x*_Sb_) (E_gGaAs_−E_gGaAsSb_) in differential strained (pseudomorphic) GaAs_1-x_Sb_x_ alloy is given by the following equation [8]:

ΔE_g_(*x*_Sb_) = 1.79*x*_Sb_ − 1.54*x*^2^_Sb_ [eV] (1)

Based on equation (1), ΔE_g_(*x*_Sb_) of GaAs_0.63_Sb_0.37_ is 0.452 eV with valence band offset ratio, Q_v_ = 1.06, a value close to 1.05—as estimated by Teissier et al [8]. Moreover, it does not contradict to the estimate of Sb molar fraction of 0.34 in GaAsSb as a transition point from type I to type-II band alignment [15] as found by Johnson *et al.* Narrowing of PL is simply caused by suppression of indirect optical transitions from GaAs barrier layers to GaAsSb QW due to type-I band alignment in structures with AlGaAs barriers. A strong decrease of total PL intensity from the sample 'a' to the sample 'c' is apparently due to collection of less number of e-h pairs in GaAsSb/GaAs QW, excited at less effective active volume in 'c' as compared to 'a'. Effective active volume for 'a' is 50 + 50 nm of GaAs barrier plus 7 nm of GaAsSb QW. However, for 'c' it is only 7 nm of GaAsSb QW.

Values of FWHM, *δE* and *T*_0_ from QW structures with different barrier layers at temperature 300 and 77 K are shown in Table 2. Sharp reduction in FWHM and *T*_0_ values for Al_0.1_Ga_0.9_As barrier layers confirms the transition from type-II to type-I band lineup. As discussed earlier, the high energy slope of the PL spectrum corresponds to a decay, exp( *-E/kT*_0_), with an equivalent temperature (*T*_0_) [8]. Sharper is the decay, as in case of type I, lower is *T*_0_. The best FWHM values for 300 and 77 K are 31.4 and 17.1 meV respectively and they are larger than *kT* for corresponding temperatures. This could be attributed to small scale roughness at QW interfaces and spatial non-uniformity of Sb content in QW.

**Table 2:** FWHM, fluctuations in electrostatic potential distribution *(δE)* and equivalent temperature *(T*_0_*)* estimated from PL spectra from QW structures with different barrier materials at temperature 300 and 77 K.

Barrier layer	300 K			77 K		
	FWHM, meV	*δE*, meV	*T*_0_, K	FWHM, meV	*δE*, meV	*T*_0_, K
GaAs	59.6	17.3	336	28.4	13.2	118
Al_0.05_Ga_0.95_As	39.2	17.1	329	25.5	11.5	115
Al_0.1_Ga_0.9_As	31.4	16.5	255	17.1	10.3	78.7

The above fact is also confirmed through a 3^rd^ set of growth, one sample with single QW and two samples with double QW structures. The purpose of this set was to mimic the QW design of a typical VCSEL device in order to achieve stronger overlap of electron-hole wave function. A single QW cross section is as follows: A 300 nm thick GaAs buffer layer, was followed by a sequence of 50 nm thick Al_0.3_Ga_0.7_As cladding layer, 30 nm thick Al_0.15_Ga_0.85_As waveguide layers, a 3 nm thick GaAs barrier layer, a 7 nm thick GaAsSb QW, a 3 nm thick GaAs barrier layer, a 30 nm thick Al_0.15_Ga_0.85_As waveguide layer and a 50 nm thick Al_0.3_Ga_0.7_As cladding layer and terminated with a 30 nm thick GaAs cap layer on top of the structure. The sequence consisting of 3 nm thick GaAs barrier layer, 7 nm thick GaAsSb QW and 3 nm thick GaAs barrier layer was repeated twice for one with double QW structure and GaAs spacer. For the other double QW structure, a 7.6 nm thick Al_0.15_Ga_0.85_As spacer was inserted in between above mentioned twice repeated QW sequence. All 3 samples were grown at 475 °C.

**Figure 5:**
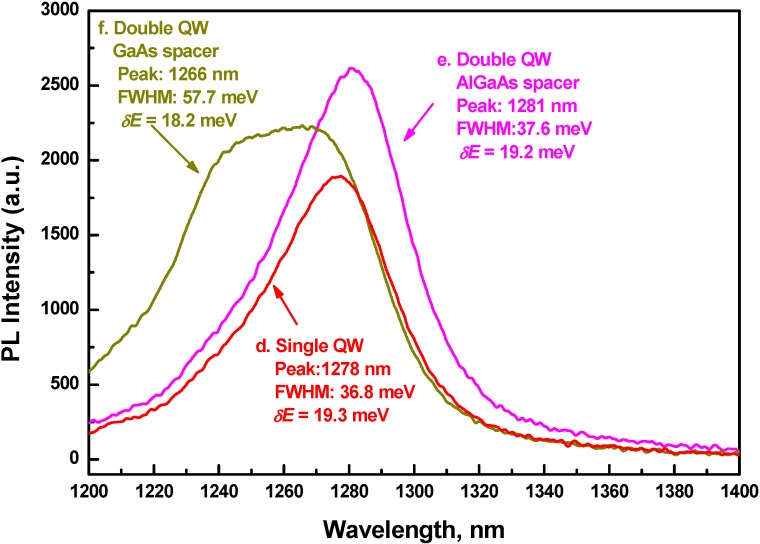
RT PL spectra from QW structures—two double QW and one single QW—with thin GaAs (3 nm) barrier layers embedded in 50 nm thick Al_0.3_Ga_0.7_As cladding and 30 nm thick Al_0.15_Ga_0.85_As waveguide layers, thus mimicking typical active layers in a VCSEL.

PL of one with thin GaAs barrier and single QW is shown as curve 'd' in Figure 4 for side by side comparison with those with thicker barriers. It clearly shows blue shift of peak and narrower linewidth with respect to samples of either curve 'a' or curve 'b'. This means that reduction of barrier thickness, from 50 nm as in a typical PL QW structure to 3 nm as in a typical VCSEL QW structure, results in switching from type-II to type-I, or at the least, from type-II to a flat conduction band lineup.

Room temperature PL spectra of all three structures in set 3 are shown in Figure 5. In spite of scattering of line position, low energy slope of the curves look similar. Fluctuations in electrostatic potential distribution, *δE*, range between 18.2 and 19.3 meV. Shape and linewidth of two structures (Curves 'd' and 'e')—one with single QW and GaAs spacer and the other with double QW and Al_0.15_Ga_0.85_As spacer—obviously do not change with number of QW. FWHM value of PL line for two of these samples is 37.2 ± 0.4 meV. This value is close to one for single QW with type-I band alignment (Table 2, structure with Al_0.1_Ga_0.9_As cladding layers, FWHM 31.4 meV). On the other hand, shape of PL line of the structure with double QW and separated by GaAs spacer (curve 'f') is completely different. Growth conditions for both type of structures, *i.e.*, one with GaAs spacer and the other with Al_0.15_Ga_0.85_As spacer, are similar. Taking into account low value of fluctuations in electrostatic potential distribution, *δE*, we should not expect compositional disorder in QW material as the main reason for distortion in PL line. We can speculate, however, that the PL line (curve 'f') consists of superposition of two lines separated approximately by 18 nm (18 meV). Total thickness of GaAs And GaAsSb at double QW structure with GaAs cladding layers is 26 nm—too large for any electron energy quantization. The PL spectrum represents a superposition of direct and indirect optical transitions in a system with delocalized electrons, as it was discussed earlier for the first set of GaAsSb/GaAs QW structures. Nevertheless, epitaxial growth of double QW separated by 6 nm thick GaAs layer or 13.6 nm thick GaAs/AlGaAs layers was successful, without any evidence of strain relaxation due to lattice mismatch. Structures with 3 QWs grown at similar conditions did not show any PL signal, most possibly due to strain relaxation.

**Figure 6:**
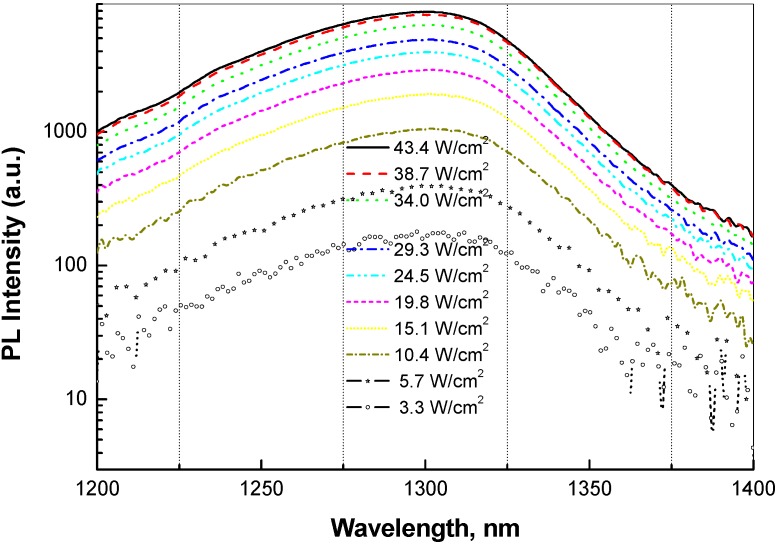
PL spectra from sample of curve 'a' shown in Figure 4 under increasing laser excitation intensity from 3.3 W/cm^2^ to 43.4 W/cm^2^.

Neither of three samples of set 2 did exhibit any blueshift of peak in RT PL under varying excitation intensity. Figure 6 shows PL spectra of curve 'a' sample—a type-II QW structure—under increasing laser excitation intensity from 3.3 W/cm^2^ to 43.4 W/cm^2^. Chiu *et al.* [17], Dinu *et al.* [9] and many others have clearly noticed blueshift over this range of excitation intensity. In contrast to what others have observed, increasing excitation changed neither shape nor position of our PL lines. Total absence of blue shift at room temperature could be attributed to negligible effect of band-bending and band-filling, because of good quality GaAsSb active material, with lower electrostatic potential fluctuations as a result of optimized growth conditions. Similar conclusion can safely be made about fluctuations in electrostatic potential caused by spatial non-uniformity of Sb content in GaAsSb QW because of miscibility gap and Sb segregation driven by lattice mismatch. In essence, these effects are not due to intrinsic properties of GaAsSb, but rather, due to un-optimized growth conditions and technological limitations. It is also possible, however, that blue shift due to band bending and band filling may have been compensated by red shift due to renormalization of QW band gap at high carrier density. It is possible that a blue shift will take place at much higher excitation intensity and also at low temperature.

## 4. Conclusions

We conclude that GaAsSb/GaAs QW with thick GaAs barriers (at least 50 nm) and with an Sb mole fraction of ~0.36 has a weak type-II band alignment with Q_V_ near 1.06. Optical transitions typically occur between weakly localized electrons and strongly localized holes. Optical properties of the QW are strongly affected by growth conditions. An FWHM of ~60 meV in RT PL indicates low fluctuation in electrostatic potential distribution (*δE* < 20 meV). It means that under optimal growth conditions, factors such as non-uniform distribution of Sb caused by miscibility gap, Sb segregation on interfaces or even internal mechanical strain play a minor role in shaping the optical properties of antimonide materials. Unoptimized growth may misrepresent the facts observed, and in turn, skew the logical conclusions. The properties of quantum wells with wider PL are typically a result of technological limitations rather than intrinsic properties of the material. We found optimal MBE growth temperature of GaAsSb QW to be 470 ± 10 °C. The maximum wavelength one could reach, with fairly intensive PL, for a 7 nm thick GaAsSb/GaAs QW structure without strain compensation, is approximately 1330 nm. Total absence of blue shift of RT PL lines under varying excitation intensity indicates that the effect of band bending and band filling in optimally grown GaAsSb material is negligible.

Thin GaAs barrier is typically preferred in GaAsSb based 1.3 micron VCSEL structure to achieve higher optical gain through type-I band alignment and stronger overlap of electron-hole wave function. However, there is a trade-off in using thin GaAs barrier and GaAsSb QW. Thinning the barrier tends to blueshift the PL line, for which more Sb needs to be incorporated in QW to compensate the blue shift. Addition of more Sb, in turn, increases mechanical strain, increases fluctuation in electrostatic potential, and above all, reduces PL intensity. So there is a potential optimum limit of maximum wavelength, which GaAsSb material can help achieve without strain compensation. Thus, the VCSEL designer must pay close attention to the QW structure in terms of PL peak and DBR resonance by considering key factors such as blueshift of PL line resulting from transition of type-II to type-I band alignment upon slight change in the QW design and red shift of PL peak resulting from shrinkage of band gap at higher temperature.
